# Electron temperature and density measurement by Thomson scattering with a high repetition rate laser of 20 kHz on LHD

**DOI:** 10.1038/s41598-022-19328-9

**Published:** 2022-09-06

**Authors:** H. Funaba, R. Yasuhara, H. Uehara, I. Yamada, R. Sakamoto, M. Osakabe, D. J. Den Hartog

**Affiliations:** 1 National Institute for Fusion Science, National Institutes of Natural Sciences, Toki, Gifu 509-5292 Japan; 2 Department of Physics, University of Wisconsin-Madison, Madison, WI 53706-1390 USA

**Keywords:** Plasma physics, Magnetically confined plasmas

## Abstract

Thomson scattering measurements with a high-repetition-rate laser have commenced in the Large Helical Device. As an example of the fast phenomena captured by this diagnostic system, measurements at a 20 kHz repetition-rate in hydrogen pellet-injected plasmas are presented. Signal processing methods for this measurement have been developed and electron temperature profiles with almost 70 spatial points were evaluated at time intervals of 50 $\upmu$s. After Raman scattering calibration, electron density profiles were derived. Fast changes in the electron temperature and density profiles within 1 ms were observed.

## Introduction

For magnetically confined fusion devices, electron temperature ($T_{\mathrm{e}}$) and electron density ($n_{\mathrm{e}}$) are fundamental parameters of plasmas. Thomson scattering diagnostics is a widely adopted measurement method of $T_{\mathrm{e}}$ and $n_{\mathrm{e}}$ in fusion plasma devices^1–17^ because of the reliability of the results and no perturbation for the plasma. Since the cross-section of Thomson scattering, $\sigma _{T}$, is almost $6.65\times 10^{-29}$ m$^{2}$, pulsed lasers with an energy of a few joules are usually used for this measurement. The Salpeter parameter, $\alpha$, determines whether the scattering is non-collective or collective. $\alpha$ is defined by $\alpha \equiv 1 / (|\varvec{k}|\lambda _D)$, where $\varvec{k}$ and $\lambda _D$ are the scattering wavenumber and Debye length, respectively. When $\alpha \ll 1$, the scattering is non-collective. In this case, the spectrum of the scattered light reflects $T_{\mathrm{e}}$ and the intensity is related with $n_{\mathrm{e}}$. It is possible to obtain a spatial profile of $T_{\mathrm{e}}$ and $n_{\mathrm{e}}$ by applying the suitable geometry of laser injection and light collection optics.

In plasmas fast phenomena are frequently observed, such as magnetohydrodynamic (MHD) instabilities, solid pellet injection and so on. It is required to measure the fast change of plasma parameters for understanding of these phenomena. In order to measure the temporal development of $T_{\mathrm{e}}$ and $n_{\mathrm{e}}$ during fast phenomena, a laser operation with short time intervals between pulses is used for Thomson scattering measurements. One way to realize such short intervals is to use several lasers. Three Nd:YAG (neodymium-doped yttrium aluminum garnet) lasers are used in the Thomson scattering system^1,2^ in the Large Helical Device (LHD)^18,19^. For a pellet-injected plasma, two of them were pulsed with a short interval of 200 $\upmu$s^20^. For the same purpose, Thomson scattering diagnostic systems with high-repetition-rate lasers have been developed in W7-X with 12 burst pulses on a 5 Hz basis^21^. Profiles of $T_{\mathrm{e}}$ and $n_{\mathrm{e}}$ were obtained with a time interval of 100 $\upmu$s during the injection process of a cryogenic $\hbox {H}_2$ pellet. Multi-pass Thomson scattering (MPTS) is one of the methods to increase an effective signal intensity of the scattered light. At the same time, with this method it is also considered that a quite fast time resolution is achieved, since the interval of multi-pass signals is shorter than 100 ns in the case of the Thomson scattering system in GAMMA10/PDX^22,23^. The double-pass intracavity ruby laser system was developed in TEXTOR and a train of 20 laser pulses with 10 kHz were produced^24^. A laser which has a repetition-rate of the order of 1 kHz is suitable for the measurement in magnetically confined plasma devices which have a duration of a few tens of milliseconds. In the Thomson scattering system in VEST, $T_{\mathrm{e}}$ and $n_{\mathrm{e}}$ at ten time points are measured during a typical plasma duration of about 20 ms, using a laser with a repetition-rate of 1 kHz^17^. Recently, Thomson scattering measurements with a high-repetition-rate Nd:YAG laser started in LHD. This laser was newly developed by a collaboration of National Institute for Fusion Science (NIFS) and the University of Wisconsin-Madison, based on a “pulse-burst” laser system^25–27^ and can be operated with a repetition frequency up to 20 kHz. Temporal development of the spatial profiles of $T_{\mathrm{e}}$ were derived and $n_{\mathrm{e}}$ profiles were also evaluated after the density calibration by Raman scattering. A pulse-burst laser system for Thomson scattering measurement was also developed in the University of Tennessee^28^.

In this study, signal processing from the data acquisition to the evaluation of $T_{\mathrm{e}}$ and $n_{\mathrm{e}}$ with a high-repetition-rate laser is established. This diagnostic is applied for a plasma with solid hydrogen pellet injection as one of the fast transient phenomena in plasmas. Then it is intended to observe fast changes in the $T_{\mathrm{e}}$ and $n_{\mathrm{e}}$ profiles with a temporal resolution of 50 $\upmu$s.

This paper consists of three major sections: Introduction, Results, and Summary before the Methods section. The Results section is divided into four sub-sections as follows: (1) “Experimental setup”, (2) “Electron temperature profiles in a pellet-injected plasma”, (3) “Raman scattering calibration”, and (4) “Electron density profiles”.

(1) The “Experimental setup” consists of three parts of the “Thomson scattering diagnostic system in LHD”, “The new high-repetition-rate Nd:YAG laser”, and “Switched-capacitor-type fast digitizers” which describe the LHD Thomson scattering system, a high-repetition-rate Nd:YAG laser and the fast digitizers for it , respectively. In sub-section (2) “Electron temperature profiles in a pellet-injected plasma”, abrupt changes in a plasma are described in the sub-subsection “Plasma parameters of a pellet-injected plasma”. Signals of the Thomson scattering diagnostics and their time-integration are shown in “Signal processing for fast digitizers”. $T_{\mathrm{e}}$ profiles in the pellet-injected plasma are presented in “Electron temperature profiles” as an example of the fast change of $T_{\mathrm{e}}$. (3) The “Raman scattering calibration” sub-section describes the electron density calibration process and the results of the calibration. In (4) “Electron density profiles”, the fast change of $n_{\mathrm{e}}$ profiles in the pellet-injected plasma is shown and the validity of the measured $n_{\mathrm{e}}$ is discussed. The Summary section is a brief synopsis of this work. The Methods section contains a description of LHD.

## Results

In this section, at first the experimental setup is described. The new laser and fast digitizer boards are explained in brief. Then the data processing methods and an example of fast temporal development of $T_{\mathrm{e}}$ are shown. In order to derive $n_{\mathrm{e}}$, the results of Raman scattering calibration are described. Lastly, the results of $n_{\mathrm{e}}$ profiles by the new laser are shown for the first time.

### Experimental setup

#### Thomson scattering diagnostic system in LHD

The Thomson scattering diagnostics system in LHD consists of the following components: lasers and the laser transfer and injection optics into the plasma, the collection and transfer optics of scattered light, the detection system of the spectrum by polychromators, the analog-to-digital conversion (ADC) of the signals, and the analysis system of $T_{\mathrm{e}}$ and $n_{\mathrm{e}}$. Nd:YAG lasers are used and the laser light is transferred by mirrors to LHD for almost 40 m. The laser pulses are injected horizontally into the equatorial plane of LHD and backscattering light is observed. The scattering angle is almost 167$^{\circ }$ at the center of the plasma. The Salpeter parameter $\alpha$ is $\alpha \ll 1$ even in low $T_{\mathrm{e}}$ and high $n_{\mathrm{e}}$ conditions, for example, $\alpha \simeq 1.1\times 10^{-2}$ for $T_{\mathrm{e}}= 100$ eV and $n_{\mathrm{e}}= 10^{20}$ m$^{-3}$. Therefore, a non-collective scattering condition is applied. The scattered light is collected and focused on the ends of optical fibers by a large concave mirror coated in gold. Then the light is transferred by the optical fibers to the inlets of the polychromators.

The main Thomson scattering system in LHD has three Nd:YAG lasers, one of which is operated at 30 Hz and the other two at 10 Hz. The laser pulses can be injected into the plasma simultaneously or separately. The number of spatial positions is 144 for the main system. A polychromator consists of five or six filter channels for spectroscopic measurement of the scattered light. In the main system, charge integration type ADCs, FASTBUS, are used.

In addition to the main system, a new Nd:YAG laser with a high-repetition-rate and fast digitizers of the switched-capacitor-type are newly installed. An output signal of one polychromator channel is amplified and separated in two. One of them is detected by the FASTBUS system, and the other output is used for checking of the signals by oscilloscopes or connected to the newly installed digitizers.

#### The new high-repetition-rate Nd:YAG laser

The new high-repetition-frequency Nd:YAG laser in LHD was developed under the collaboration of NIFS and the University of Wisconsin-Madison, based on a “pulse-burst” laser system. This laser can be operated with two kinds of repetition frequencies, one of which is 1 kHz with 30 laser pulses and the other is 20 kHz with 100 laser pulses. Therefore, the time range of the measurement is 30 ms and 5 ms for the 1 kHz and 20 kHz cases, respectively. The typical energy of the laser pulse is 1.6 J for the 1 kHz operation and almost $1\sim 1.2$ J for the 20 kHz operation. The signals of the laser pulses during the operation are monitored in the 1 kHz case, while they are not obtained yet in the 20 kHz case.

#### Switched-capacitor-type fast digitizers

The scattered light from the plasmas is detected by polychromators and the AC component of the signal is amplified. Then the signals are acquired by the ADC. Since the frequency of the high-repetition-rate laser is up to 20 kHz, it is necessary to acquire the data with an interval of 50 $\upmu$s. The charge-integration type detector is not suitable for the measurement with such a short interval, because several time gates for the signal frame and some background frames are needed. Therefore, new multi-channel fast digitizer boards of a switched-capacitor-type, TechnoAP APV85G32L, were installed. One board of this digitizer has 32 channels of inputs and acquires data with a sampling frequency of 1 GS/s. The minimum read-out time which is needed to convert analog to digital in these switched-capacitor-type digitizers, is shorter than 50 $\upmu$s. The number of spatial positions measured by this new digitizer system is almost 70 with the 12 boards.

This fast digitizer includes 4 DRS4 (Domino Ring Sampler)^29^ chips which are switched-capacitor arrays. Each channel has 1024 storage cells. In the 1 kHz operation of the laser, the length of the data is 1024 points, while it becomes 256 points in the 20 kHz operation. The data which are acquired by the DRS4 need some corrections, such as the cell amplitude calibration which corrects the difference of the amplitude of the capacitors, the peak correction of which removes a few spikes with one or two points appearing at the same timing among the DRS4 channels, and so on. The peak correction can be made by interpolation.

### Electron temperature profiles in a pellet-injected plasma

#### Plasma parameters of a pellet-injected plasma

**Figure 1:**
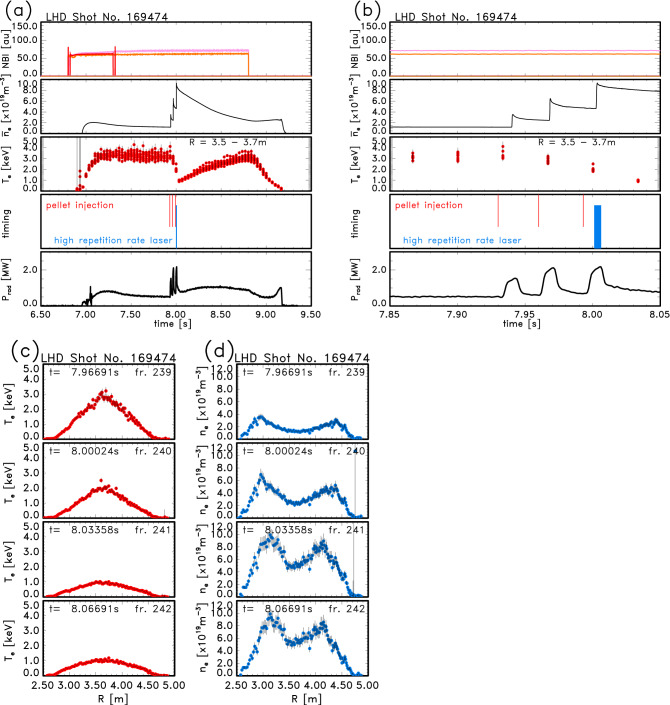
Some plasma parameters in a solid pellet-injected plasma. The temporal development of NBI heating pulses, line-averaged electron density (${\overline{n}}_{\mathrm{e}}$), $T_{\mathrm{e}}$, timings of pellets (red) and laser pulses of the high-repetition-rate laser (blue), and radiation power ($P_{\mathrm{rad}}$) and the $T_{\mathrm{e}}$ and $n_{\mathrm{e}}$ profiles in a pellet-injected plasma in LHD. These $T_{\mathrm{e}}$ and $n_{\mathrm{e}}$ profiles are measured by a laser with a repetition frequency of 30 Hz. (**a**) the whole time of the plasma, (**b**) the expanded time around the pellet injection, (**c**) $T_{\mathrm{e}}$ profiles, and (**d**) $n_{\mathrm{e}}$ profiles.

In this subsection, methods of the signal processing for the switched-capacitor-type digitizers and the evaluation of $T_{\mathrm{e}}$ are described. As an example of fast changes of plasma parameters, a hydrogen pellet-injected plasma is shown. Figure 1a shows the temporal development of some plasma parameters such as neutral beam injection (NBI) heating pulses, line-averaged electron density (${\overline{n}}_{\mathrm{e}}$) which was measured by a far infrared (FIR) interferometer, $T_{\mathrm{e}}$ around the plasma center by Thomson scattering with a 30 Hz laser, timings of pellets and the new laser, and radiation power ($P_{\mathrm{rad}}$) in an LHD plasma where three solid hydrogen pellets were injected by the LHD pellet injector^30^. The abscissa of Fig. 1b is an expansion of Fig. 1a around the pellet injection timings. Since the timing lines of the pellets (red) are based on signals from the pellet injection system, they are almost 10 ms earlier than the actual injection timings. The laser timings of the high-repetition-rate laser are also shown in Fig. 1a, b. 100 laser pulses were injected within 5 ms around $t = 8.0$ s ($t = 8.00120 \sim 8.00615$ s). The timing of the first laser pulse is just before the actual injection of the third pellet. The temporal development of the spatial profile of $T_{\mathrm{e}}$ is shown in Fig. 1c and that of $n_{\mathrm{e}}$ profiles is shown in Fig. 1d. The abscissa, *R*, is the major radius of the torus plasma. These $T_{\mathrm{e}}$ and $n_{\mathrm{e}}$ profiles are measured by the Thomson scattering with a laser of a repetition frequency of 30 Hz. The $n_{\mathrm{e}}$ profile at $t = 7.96691$ s has already become hollow due to the first pellet.

In Fig. 1c, $T_{\mathrm{e}} \simeq 3$ keV in the center region at $t = 7.96691$ s is the timing before the second pellet reached the plasma. The timing of 8.00024 s is just before the actual injection of the third pellet. Although the $T_{\mathrm{e}}$ and $n_{\mathrm{e}}$ significantly changed between $t = 8.00024$ and 8.03358 s, detailed change cannot be known from this laser frequency. It is considered that the profiles mainly changed within 1 ms from the previous results. After the pellet injection, a plasmoid component is formed^31,32^ in the background plasma. The pellets are injected into a horizontally elongated cross-section of the plasma. Since the difference of the toroidal angle between the pellet injection and the Thomson scattering diagnostics is $\frac{1}{5}\pi$, there may be a possibility that the plasmoid component can be measured by the Thomson scattering system. The timings when such a plasmoid may exist were not included in Fig. 1d.

#### Signal processing for fast digitizers

**Figure 2:**
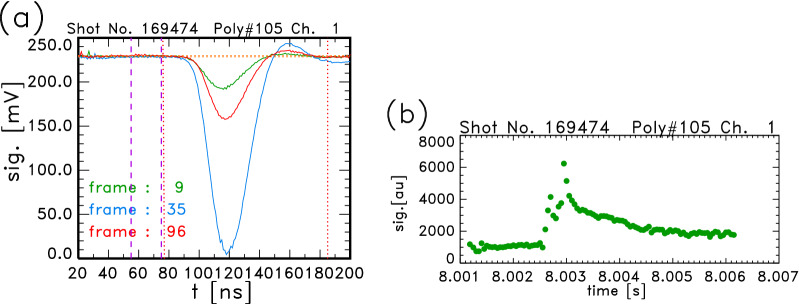
Signals of Thomson scattering measurement. (**a**) Examples of the signals of Thomson scattering in a pellet-injected plasma. Frames Nos. 9, 35 and 96 correspond to $t = 8.00165, 8.00295$ and 8.00600 s, respectively. (**b**) Temporal development of an integrated signal. The abscissa represents the same time as in Fig. 1.

Figure 2a shows some signals of Thomson scattering measurement which were acquired by the new switched-capacitor-type digitizers, APV85G32L, in the same pellet-injected plasma of Fig. 1. Cell amplitude calibration and a peak correction were made. These signals were detected from polychromator No. 105 (Poly#105), which observes at the major radius position of $R = 4.376$ m. This position is located almost in the middle of the outside of the magnetic axis. The usual polychromator has five spectral channels for the Thomson scattering measurement. The data of channel 1, which is the closest channel to the laser wavelength, are shown here. The frame number *i* corresponds to the signal by the ($i+1$)th laser pulse. The frames Nos. 9, 35 and 96 correspond to $t = 8.00165, 8.00295$ and 8.00600 s, respectively.

In order to derive $T_{\mathrm{e}}$ and $n_{\mathrm{e}}$, precise evaluation is needed in the following processes. These are the evaluation of the background level, the determination of the signal gate timing, the time-integration of the signals, and the subtraction of stray light. The background level is derived by averaging the data just before the signal, which is indicated by the region between the purple broken lines in Fig. 2a. The gate timing where the time integration is made is shown between the red dotted lines. The width of this gate is 110 ns and the timing is adjusted for each polychromator. The time-integration is made by a simple summation of the data in this gate timing, as the first step of the analysis. The stray light component is estimated from the signal, which is acquired without the plasma, and it is subtracted after the integration. In the case of Fig. 2a, the stray light component is quite small. Figure 2b shows the temporal development of the signal intensity of channel No. 1 of Poly#105. The value of the signal is obtained from the product of the voltage and time in the units of [mV] and [ns], respectively. On the other hand, it is found that the estimated number of detected photons in one channel of the polychromator may be in a similar order of the value of the integrated signal. Then this value is considered to be related with the number of photons and the unit here is expressed by [au]. The abscissa represents the same time in Fig. 1. The relative energy of each laser pulse is estimated from the signal of the laser pulse for the 1 kHz operation. However, since it is not yet observed in the 20 kHz operation because of the short time range of 255 ns, constant energy is assumed for the 20 kHz case. The intensity increased after frame No. 28, which corresponds to $t = 8.00260$ s, and it became quite large at frame No. 35 ($t = 8.00295$ s), but saturation of the signal was not found.

#### Electron temperature profiles

The electron temperature is evaluated by the $\chi ^2$-method which minimize the following $\chi ^2$,

1 $$\begin{aligned} \chi ^2 = \sum _i w_i (x_i - \lambda _c s_i)^2, \end{aligned}$$

where *i* represents *i*-th channel of a polychromator and $x_i, s_i, w_i, \lambda _c$ are the signal intensity, the expected intensity for the specific $T_{\mathrm{e}}$, the weight and a constant coefficient, respectively. The error of $T_{\mathrm{e}}$ is evaluated from the $T_{\mathrm{e}}$ region which provides $\chi ^2$ under $\chi _{\mathrm{min}}^2 + \Delta \chi ^2$, where $\chi _{\mathrm{min}}^2$ denotes the minimum $\chi ^2$. When the shot noise is considered to be a main cause of the error, $\Delta \chi ^2 = 1$ can be used with $w_i$, which corresponds to the inverse of the magnitude of the signals. For the first analysis, constant $w_i = 0.001$ is assumed for all channels in order to avoid that the magnitude of weight becomes unstable when the signal is small. This weight is chosen because the value of the integrated signal is usually in the range of $100\sim 3000$. The results of $T_{\mathrm{e}}$ are similar to those from the 30 Hz laser which are evaluated with $w_i$, considering the shot noise and fluctuation of the background light. The time-integrated signals are used as $x_i$. In order to confirm the validity of $T_{\mathrm{e}}$ profiles, the signals and temporal evolution of $T_{\mathrm{e}}$ in almost constant $T_{\mathrm{e}}$ plasmas were checked.

**Figure 3:**
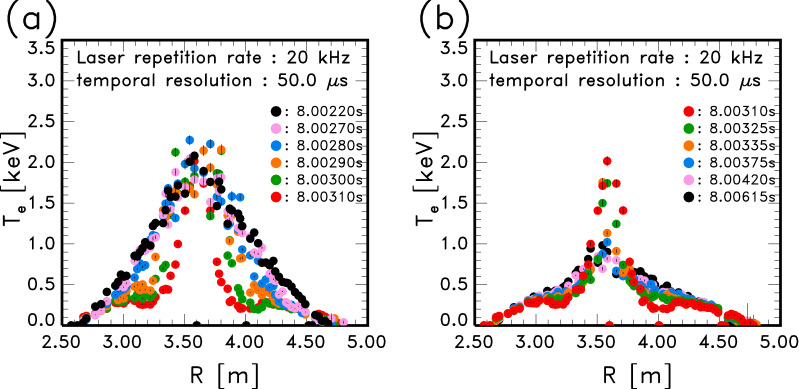
Temporal development of $T_{\mathrm{e}}$ profiles in a pellet-injected plasma. (**a**) $t = 8.00220 \sim 8.00310$ s, (**b**) $t = 8.00310 \sim 8.00615$ s.

Figure 3 shows the temporal development of the $T_{\mathrm{e}}$ profiles which were obtained in the same plasma as in Fig. 1. The position of the magnetic axis in a vacuum was $R = 3.60$ m. The number of spatial positions was almost 70 in these profiles. Some selected timings when the $T_{\mathrm{e}}$ profiles were clearly distinguishable are shown here, although the actual time resolution was 50 $\upmu$s. The $T_{\mathrm{e}}$ profile at the first timing $t = 8.00220$ s (*black circles*) was almost the same as that at $t = 8.00024$ s in Fig. 1c. The pellet was injected from outside of the torus. $T_{\mathrm{e}}$ decreased from the outside of the plasma, while $T_{\mathrm{e}}$ at the center region still remained high until $t = 8.00310$ s. Thus, the $T_{\mathrm{e}}$ profile changed within 1 ms. It was found that the time interval of 50 $\upmu$s had enough temporal resolution. From the results of $T_{\mathrm{e}}$ profiles between $t = 8.00310$ and 8.00615 s in Fig. 3b, $T_{\mathrm{e}}$ at the center decreased and $T_{\mathrm{e}}$ around $R \simeq 3.2$ and 4.0 m gradually recovered.

### Raman scattering calibration

**Figure 4:**
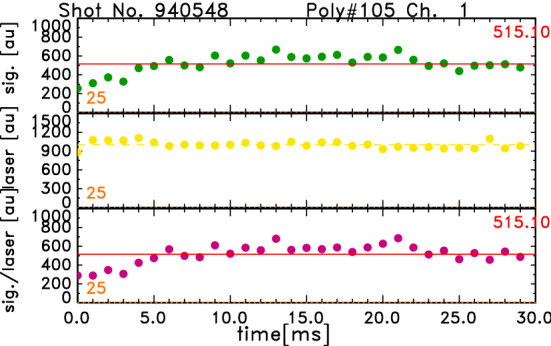
Data of Raman scattering. The *green data* are the temporal development of the integrated signal of Raman scattering in the 1 kHz operation of the laser. The *yellow data* are those of the laser signal. The red line indicates the average of these data. The *magenta data* are the Raman data which are normalized by the laser power.

The results of the electron density calibration by Raman scattering for the Thomson scattering diagnostics with the high-repetition-rate Nd:YAG laser are shown in this subsection. Figure 4 shows the data of the Raman scattering calibration, which was made by filling the vacuum vessel with dry air. The data were obtained in the same channel of Fig. 2 and the laser was in the 1 kHz operation. The *green data* are the temporal development of the integrated signal of Raman scattering and the *yellow data* are those of the laser signals, which correspond to the energy of the laser pulses. The red line indicates the average of these data. The number of laser pulses used in the averaging is 30 in the 1  kHz case and 100 in the 20 kHz case, which are the numbers in one series of laser operation. The *magenta data* are the Raman data which are normalized by the laser power. The fluctuation of the laser power is not large except for the first pulse. A few data just after the start of the laser become small, although the laser power is not small. The reason for this is not clear at present. The scattering of these data may be larger than the actual Raman data, since the noise components which were caused by the digitizers remained, although corrections were made. However, it is considered that the effects of such noises may be small in high densty cases like pellet injection experiments. In this paper, the temporal integration of signals is made simply by a summation as the first step of the analysis. However, it is considered that the effects of such noise components can be removed by signal processing. For the 20 kHz operation, the laser power is not obtained and it is assumed to be constant in the analysis.

**Figure 5:**
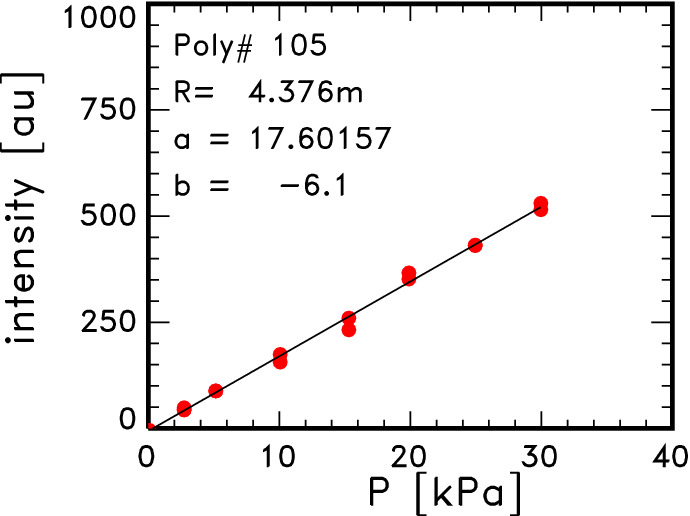
Results of Raman scattering calibration. Relation of the averaged Raman signal intensity and pressure. *a* and *b* are the gradient and y-intercept of the linear approximation.

Figure 5 shows the dependence of the averaged Raman signal intensity, which is shown by the red line in Fig. 4, on the pressure, *P*. The Raman data were obtained under $P \simeq 3\times 10^{4}$ Pa. As the value of y-intercept of the linear approximation, *b*, is small enough, the gradient of this relation, *a*, is used for the density calibration. The intensity of Raman scattered light in the spectral channel No. 1, $S_1^R$, is expressed by

2 $$\begin{aligned} S_1^R = a P + b \simeq a P = a n_0 k T_0, \end{aligned}$$

where $n_0, k$, and $T_0$ are the neutral density of the air, the Boltzmann constant and the room temperature, respectively.

The signal intensity of Thomson scattering for the spectral channel No. *i*, $S_i^T$, and that of Raman scattering for the spectral channel No.1 , $S_1^R$, are expressed as follows^33^.

3 $$\begin{aligned}&S_i^T = AP_Ln_{\mathrm{e}}\int \sigma ^T(T_{\mathrm{e}}, \lambda ) f_i(\lambda )d\lambda \frac{dI^T}{d\Omega } \end{aligned}$$

4 $$\begin{aligned}&S_1^R = AP_L\left[ n^{\mathrm{N}}\sum _J w_J^{\mathrm{N}}\sigma _J^{\mathrm{N}}(\lambda _J^{\mathrm{N}})f_1(\lambda _J^{\mathrm{N}}) + n^{\mathrm{O}}\sum _J w_J^{\mathrm{O}}\sigma _J^{\mathrm{O}}(\lambda _J^{\mathrm{O}})f_1(\lambda _J^{\mathrm{O}}) \right] \frac{dI^R}{d\Omega }, \end{aligned}$$

Here *A* is a common coefficient in Thomson and Raman scattering, $P_L$ is the energy of the laser pulse, and $\lambda$ is the wavelength. In Eq. (3), $\sigma ^T$ is the cross-section of Thomson scattering, $f_i$ is the spectral responsibility of channel No.*i*, and $\frac{dI^T}{d\Omega }$ is the angular distribution of Thomson light. In Eq. (4), *J* is the initial rotational-angular-momentum quantum number, $w_J^{N,O}$ is the population of the initial rotational state, and $\sigma _J^{N,O}$ is the cross-section of the anti-Stokes rotational $J^{'} = J - 2$ Raman transition. ’N’ and ’O’ represent nitrogen and oxygen, respectively. $\frac{dI^R}{d\Omega }$ is the angular dependence of the differential cross-section of the quadrupole Raman transition.

**Figure 6:**
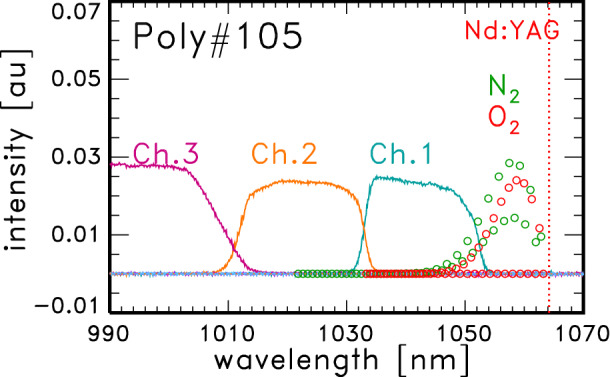
Raman coefficients and the polychromator channels. Wavelength dependence of the transparency of the filters in polychromator No. 105 and cross-sections of the Raman scattering for molecular nitrogen (*green circles*) and oxygen (*red circles*). The red dotted line at 1064 nm shows the wavelength of the Nd:YAG laser.

Figure 6 shows the wavelength dependence of the transparency of the filters in Poly#105 and the cross-sections of the Raman scattering for molecular nitrogen (green circle) and oxygen (red circle). The Raman signal is mainly detected by channel No. 1 (cyan curve). By using the ratio of nitrogen and oxygen, $r^{\mathrm{N}} = n^{\mathrm{N}} / n_0$ and $r^{\mathrm{O}} = n^{\mathrm{O}} / n_0$, respectively, the effective Raman cross-section, $\sigma _{\mathrm{eff}}^R$ is defined as

5 $$\begin{aligned} \sigma _{\mathrm{eff}}^R = r^{\mathrm{N}} \sum _J w_J^{\mathrm{N}}\sigma _J^{\mathrm{N}}(\lambda _J^{\mathrm{N}})f_1(\lambda _J^{\mathrm{N}}) + r^{\mathrm{O}} \sum _J w_J^{\mathrm{O}}\sigma _J^{\mathrm{O}}(\lambda _J^{\mathrm{O}})f_1(\lambda _J^{\mathrm{O}}), \end{aligned}$$

and Eq. (4) is written as

6 $$\begin{aligned} S_1^R = AP_L n_0 \sigma _{\mathrm{eff}}^R \frac{dI^R}{d\Omega } = a n_0 k T_0. \end{aligned}$$

Here the relation of Eq. (2) is used. The total intensity of Thomson scattering signals in a polychromator is expressed by

7 $$\begin{aligned} \sum _iS_i^T = AP_Ln_{\mathrm{e}} \sum _i\int \sigma ^T(T_{\mathrm{e}}, \lambda ) f_i(\lambda )d\lambda \frac{dI^T}{d\Omega }. \end{aligned}$$

Then $n_{\mathrm{e}}$ is evaluated by Eq. (7) where *A* is derived from Eq. (6). The difference of laser power between the 1 kHz and 20 kHz operations is included in the Raman coefficients for the electron density calibration.

### Electron density profiles

**Figure 7:**
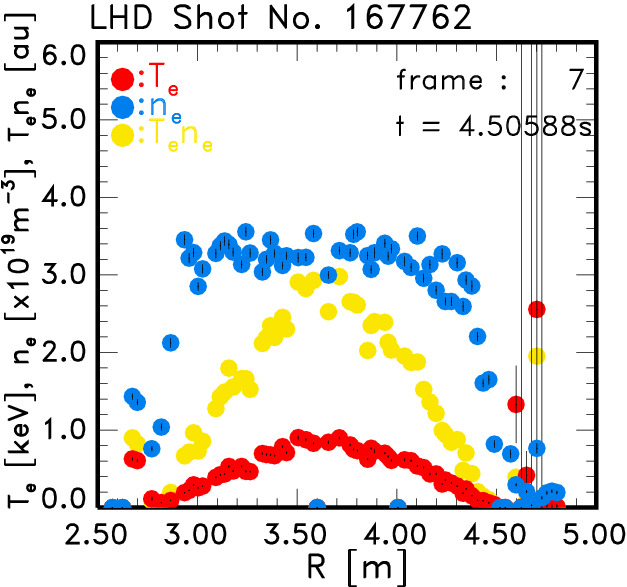
Confirmation of the validity of the electron density measurement. An example of $n_{\mathrm{e}}$ profiles in a plasma with a relatively flat shape. The data were acquired in the 1 kHz opertion. $n_{\mathrm{e}}$ (*blue circles*), $T_{\mathrm{e}}$ (*red circles*), and the product of $n_{\mathrm{e}}T_{\mathrm{e}}$ (*yellow circles*) are shown.

In this subsection the $n_{\mathrm{e}}$ profiles, which are calculated with the Raman calibration data, are shown. In order to confirm the validity of the calibration, the $n_{\mathrm{e}}$ profile of a plasma with $n_{\mathrm{e}}\simeq 3\times 10^{19}$ m$^{-3}$ is shown in Fig. 7 as *blue* *closed circles*. The $T_{\mathrm{e}}$ profile (*red circles*) and the product of $n_{\mathrm{e}}T_{\mathrm{e}}$ (*yellow circles*) are also shown in the same figure. The data were acquired in the 1 kHz operation of the laser. The $n_{\mathrm{e}}$ profile in this plasma was relatively flat and almost the same $n_{\mathrm{e}}$ value and profile was also observed by the Thomson scattering measurement with the 30 Hz laser, where the $n_{\mathrm{e}}$ calibration was made independently. The line-averaged electron density by the FIR interferometer was almost $2.6\times 10^{19}$ m$^{-3}$, while the averaged value of $n_{\mathrm{e}}$ in Fig. 7 along the laser line was almost $2.8\times 10^{19}$ m$^{-3}$. Here only the effect of the shot noise is considered as an error of $n_{\mathrm{e}}$, since the estimation of the scattering of the Raman data may be too large, because of the noises from the digitizers.

**Figure 8:**
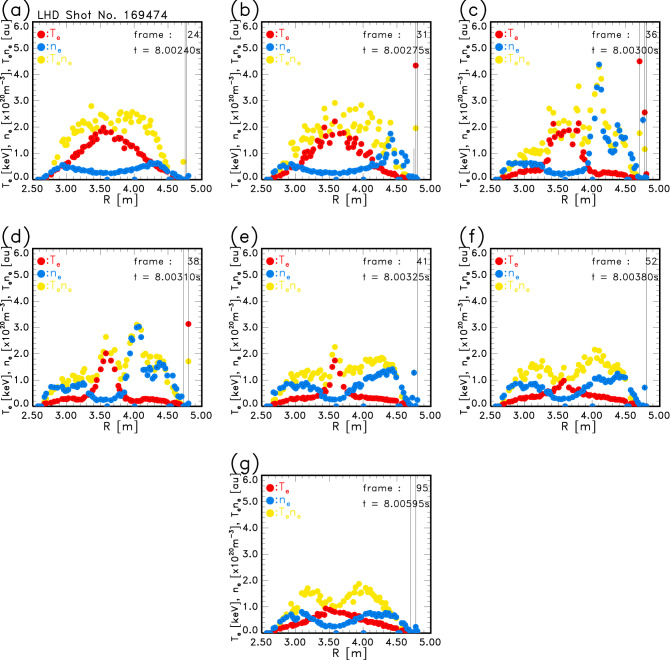
Temporal development of the $n_{\mathrm{e}}$ profile of the pellet-injected plasma which was measured by the 20 kHz operation of the laser. $n_{\mathrm{e}}$ (*blue circles*), $T_{\mathrm{e}}$ (*red circles*) and the product of $n_{\mathrm{e}}T_{\mathrm{e}}$ (*yellow circles*) are shown.

Temporal development of the $n_{\mathrm{e}}$ profile of the pellet-injected plasma is shown in Fig. 8a–g. The marks are the same as in Fig. 7, that is, $n_{\mathrm{e}}$, $T_{\mathrm{e}}$ and $T_{\mathrm{e}}n_{\mathrm{e}}$ are indicated by *blue*, *red* and *yellow* *closed circles*, respectively. $n_{\mathrm{e}}$ started to increase at $t = 8.00275$ s (Fig. 8b) from the outside of the torus. Since the velocity of the pellet was almost 1 km/s, it is considered that it progressed about 1 m in 1 ms. The low $T_{\mathrm{e}}$ region where $T_{\mathrm{e}}\le 0.3$ keV seemed to penetrate into the center region of the plasma. In Fig. 8c, quite high $n_{\mathrm{e}}$ was observed in the torus outer region. The pellet seemed to vanish by ablation at $t = 8.00310$ s (Fig. 8d) because the low $T_{\mathrm{e}}$ region stopped penetrating. In the spatial profiles of $n_{\mathrm{e}}$ and even in $n_{\mathrm{e}}T_{\mathrm{e}}$, asymmetry between the inner and the outer side of the magnetic axis was found during the pellet ablation time. The reason for this asymmetry is considered because these spatial profiles of $n_{\mathrm{e}}$ also contain the plasmoid component, which is formed by the pellet ablation, in addition to the background electron density. From Fig. 8e–g, the $n_{\mathrm{e}}$ profile seems to show recovery of the symmetry. A similar $T_{\mathrm{e}}$ profile with a steep gradient at the center like Fig. 8d and high $n_{\mathrm{e}}$ in the outer region, like Fig. 8c,d, are already observed^20^. However, the gradual change of the $T_{\mathrm{e}}$ and $n_{\mathrm{e}}$ within 1 ms is observed for the first time in the pellet-injected plasma in LHD.

## Summary

Thomson scattering diagnostics with a high-repetition-rate laser, which can be operated up to 20 kHz, have started in LHD. Some processes for analyzing the Thomson scattered signals with the temporal development is established, such as the determination of the background level, the gate timings for the time-integration, the temporal integration method and so on. In this first analysis, the temporal integration was made by simple summation.

$n_{\mathrm{e}}$ calibration by Raman scattering was made. The evaluated $n_{\mathrm{e}}$ profiles show valid results for plasmas with a relatively flat shape. The first results of the temporal evolution of $T_{\mathrm{e}}$ and $n_{\mathrm{e}}$ profiles in a pellet-injected plasma are shown. The penetration of the low $T_{\mathrm{e}}$ region into the center of the plasma and a quite high $n_{\mathrm{e}}$ region, which consists of the plasmoid component and background plasma, is formed outside of the torus. The temporal development of $T_{\mathrm{e}}$ and $n_{\mathrm{e}}$ within 1 ms is observed in detail by these diagnostics.

## Methods

### The Large Helical Device (LHD)

LHD is a magnetically confined fusion plasma experiment device of a heliotron-type with the poloidal period number $l=2$ and the toroidal period number $m=10$. The major radius of the magnetic axis in the vacuum $R_{\mathrm{ax}}$ is from 3.5 to 4.1, and the average minor radius, $a\simeq 0.6$ m. LHD consists of many systems, such as superconducting coil systems, power supplies, a helium refrigerator, vacuum pumping systems, plasma heating systems, plasma control systems, plasma diagnostic systems, and so on. The magnetic surfaces for plasma confinement are created by external coils which are made with a superconductor and cooled at liquid helium temperature. Therefore, plasma current is not required for plasma confinement. The plasmas are heated by electron cyclotron resonant heating (ECH), neutral beam injection (NBI), and ion cyclotron resonant heating (ICH). The plasmas are usually started by ECH. Moreover, it is possible to start them up by NBI. As control systems, the divertor system, resonant magnetic perturbation (RMP) field coils, fueling systems, and so on are installed. For fueling, hydrogen, deuterium, helium and other gases are provided by a gas-puff system. Solid cryogenic hydrogen or deuterium are injected into the plasmas by the pellet injector. Various systems for the measurement of the plasma parameters are installed, such as electron temperature and density, ion temperature, radiation power, electric potential, fluctuations, neutron yield, and diagnostics for spectroscopic measurements, impurity injection, high energy ions, measurements for the peripheral plasma, and so on. The typical parameters used in this paper are as follows. The magnetic field strength at the magnetic axis, $B = 2.75$ T, the electron temperature, $T_{\mathrm{e}}= 0.1 \sim$ a few  keV, the electron density, $n_{\mathrm{e}}= 0.5\times 10^{19}\sim$ more than $2\times 10^{20}$ m$^{-3}$.

## Data Availability

The data of $T_{\mathrm{e}}$ and $n_{\mathrm{e}}$ profiles in the figures of this paper are available from the LHD experiment data repository at https://www-lhd.nifs.ac.jp/pub/Repository_en.html after reading “Data Rights and Rules” and submitting the “LHD Data Usage and Publication Agreement” form. The other data of this study are available from the corresponding authors upon reasonable request.
